# Using Blue Intensity from drought-sensitive *Pinus sylvestris* in Fennoscandia to improve reconstruction of past hydroclimate variability

**DOI:** 10.1007/s00382-020-05287-2

**Published:** 2020-05-13

**Authors:** Kristina Seftigen, Mauricio Fuentes, Fredrik Charpentier Ljungqvist, Jesper Björklund

**Affiliations:** 1 Regional Climate Group, Department of Earth Sciences, University of Gothenburg, Gothenburg, Sweden; 2 Georges Lemaître Centre for Earth and Climate Research (TECLIM), Earth and Life Institute, Université Catholique de Louvain (UCL), Louvain-la-Neuve, Belgium; 3 Swiss Federal Institute for Forest Snow and Landscape Research WSL, Birmensdorf, Switzerland; 4 Department of History, Stockholm University, Stockholm, Sweden; 5 Bolin Centre for Climate Research, Stockholm University, Stockholm, Sweden; 6 Swedish Collegium for Advanced Study, Uppsala, Sweden

**Keywords:** Dendroclimatology, Tree-ring, Blue Intensity, Scots pine, Hydroclimate, Fennoscandia, Drought sensitivity

## Abstract

High-resolution hydroclimate proxy records are essential for distinguishing natural hydroclimate variability from possible anthropogenically-forced changes, since instrumental precipitation observations are too short to represent the whole spectrum of natural variability. In Northern Europe, progress in this field has been hampered by a relative lack of long and truly moisture-sensitive proxy records. In this study, we provide the first assessment of the dendroclimatic potential of Blue Intensity (BI) and partial ring-width measurements (latewood and earlywood width series) from a network of cold and drought-prone *Pinus sylvestris* L. sites in Sweden. Our results show that all tree-ring parameters and sites share a clear and strong sensitivity to warm-season precipitation. The ΔBI parameter, in particular, shows considerable potential for hydroclimate reconstructions, here permitting a cross-validated precipitation reconstruction capable of explaining 56% (1901–2010 period) of regional-scale warm-season high-frequency precipitation variance. Using ΔBI as an alternative to ring-width improves the predictive skill with nearly a 20 percentage points increase in explained variance, reduces signal instability over time as well as allows a broader seasonal window (May–July) to be reconstructed. Additionally, we found that earlywood BI also reflect a positive late winter through early summer temperature signal. These findings emphasize that tree-rings, and in particular wood density parameters such as from BI, are capable of providing fundamental information to advance our understanding of hydroclimate variability in regions with a cool and rather humid climate regime that traditionally has been overlooked in studies of past droughts. Increasing the spatio-temporal coverage of hydroclimate records in northern Europe, and taking full advantage of the opportunities offered by the wood densitometric properties should be considered a research priority.

**Electronic supplementary material:**

The online version of this article (10.1007/s00382-020-05287-2) contains supplementary material, which is available to authorized users.

## Introduction

The drought vulnerability of the usually cool and wet region of northern Europe was highlighted in the extreme 2018 summer drought. The prospect of future global-scale climate change modifying exposure to drought warrants the urgent need for a better understanding of regional drought dynamics, associated physical mechanisms, and the range of conditions that can be expected in the future, as a sound basis for the successful implementation of mitigation and adaptation strategies (Toreti et al. 2019; Trnka et al. 2018). This includes a robust understanding of the sensitivity of the climate system to different forcings. However, achieving such understanding is not straightforward due to the chaotic nature of intrinsic climate variability, which in the short-term, as well as on decadal and longer time scales can amplify or dampen forced trends and signals (Ljungqvist et al. 2016, 2019). These constraints present challenges in our ability to separate the role of anthropogenic forcing, natural forcing, and long-term natural variability in post-industrial hydroclimate change (Barnett et al. 2005). Establishing a baseline variability unperturbed by significant anthropogenic forcing over decadal-to-centennial time-scales therefore require a perspective that can be obtained only from pre-industrial hydroclimate information (Cook et al. 2004, 2015, 2016; Marvel et al. 2019).

Climate reconstructions over the Common Era (CE) in Scandinavia rely heavily on information derived from tree-ring data. This region is, in fact, one of the most prominent regions in the world for high-quality tree-ring based temperature reconstructions (Esper et al. 2016, 2018; Linderholm et al. 2015; St. George and Ault 2014). It is, moreover, evident from the literature that wood density of the latewood, maximum density (MXD), is particularly skillful in characterizing past temperature changes over the past one to two millennia (e.g., Björklund et al. 2019). There are numerous advantages of MXD over tree-ring width (TRW) as a climate proxy. MXD generally contains a stronger climate signal with higher signal-to-noise ratios (Ljungqvist et al. 2020a), shows less biological persistence (Esper et al. 2015) and age-related signal-muting (Konter et al. 2016), and is less influenced by stand disturbances (Rydval et al. 2018). Past hydroclimate of Fennoscandia has attracted less attention (Linderholm et al. 2010, 2018), but studies using TRW have been surprisingly successful considering the cold to mild/humid climate of the region (e.g., Drobyshev et al. 2011; Helama et al. 2009; Seftigen et al. 2015a; Seftigen et al. 2013). Considering the success of wood densitometric parameters in studying past temperatures, it is the logical next step to explore whether the density of drought-sensitive trees of the region similarly can improve or complement our understanding of past hydroclimate variability in Scandinavia. In fact, wood density of the earlywood has been shown to carry a pronounced precipitation signal in drier climates of the Iberian Peninsula, southwestern United States and Siberia (Camarero et al. 2014, 2017; Cleaveland 1986).

Wood densitometric parameters can be derived using several different measurement techniques: X-ray based densitometry, cell anatomically-based densitometry or simply from the reflection of visible light from carefully prepared wood surfaces (Björklund et al. 2019). Although these various techniques differ in measurement resolution, reliable results can be obtained at inter-annual to multi-decadal time-scales with low-cost techniques using a commercial flatbed scanner to generate image of reflected or absorbed blue light variation in wood (BI; McCarroll et al. 2002). In an exploratory study, where low cost, speed and sufficient accuracy all are important features; BI-based wood density estimations become very attractive.

**Fig. 1:**
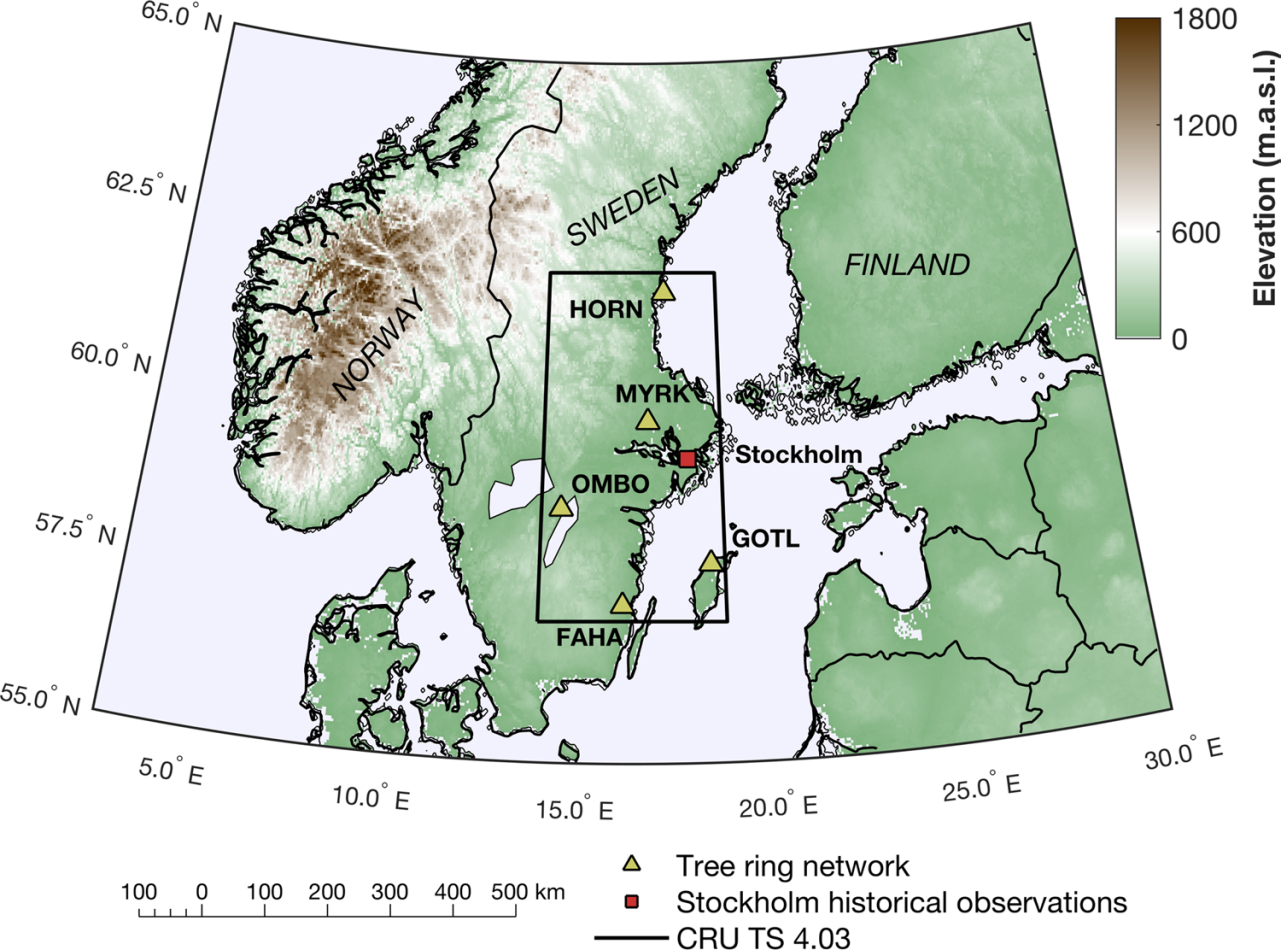
Geographical distribution of the five tree-ring chronology sites in Sweden, together with the outline of the 57–62° N/14–19° E subset of the CRU TS 4.03 product (Harris et al. 2014) and the location of the Stockholm historical weather observatory dataset used in the calibration/validation exercise

Building upon previous tree-ring based drought research in Fennoscandia (Seftigen et al. 2015a, b, 2017), this article provides the first exploration of BI (including maximum latewood BI, average earlywood BI, and delta BI) and partial ring-width measurements (earlywood and latewood widths) from a network of cool and drought-prone *Pinus sylvestris* sites in southern and central Sweden (latitudes 57°*–*62° N; Fig. 1). This study assess the dendroclimatic potential of these sub-annual parameters by evaluating the nature, strength and temporal stability of their seasonal climate signal. In particular, we are interested in whether utilizing information from this tree-ring proxy ensemble may yield more robust hydroclimate reconstructions than prior reconstructions based only on ring-width. If these parameters indeed can provide superior or complementary information, they would be a critical resource to bridge some of the current spatiotemporal data gaps of high-resolution hydroclimate proxy data across northern high latitudes, recently highlighted in the review by Linderholm et al. (2018).

## Materials and methods

### Chronology development

For our study, we selected a subset of the tree-ring network presented in (Seftigen et al. 2015a), consisting of five sites from Sweden that forms a ~ 500 km north–south transect from latitudes 57° to 62°N (Fig. 1). The annual mean temperature varies throughout the region between 2 and 7 °C. The range for the warmest month of the year, July, is 13 °C and 18 °C and for the coldest month, February, between –1 and –9 °C over the reference period 1961–1990. The mean daily maximum temperature exceeds 20 °C in most of the region during June–August, with maximum temperatures frequently exceeding 25 °C. Annual total precipitation varies throughout the region between ~ 450 and ~ 800 mm over the same reference period. The driest time occurs in late winter to spring, with one fifth of the precipitation falling in February–May. The length of the growing season varies from over 200 days in the southeast to less than 140 days in the northwest. Tree-ring width (TRW) records of Scots pine (*Pinus sylvestris* L.) at these sites is documented to be particular sensitive to May–June precipitation and were, together with additional tree-ring collections, used previously to reconstruct gridded warm-season Standardized Precipitation–Evapotranspiration Index (SPEI; Vicente-Serrano et al. 2010) for the past millennium of the Common Era (Seftigen et al. 2017).

At each site, one to two 5 mm diameter cores were extracted from approximately 20 dominant or co-dominant living Scots pine trees for BI and partial ring width analyses (Table 1). Samples from dry microsites, characterized by rocky shallow soils, were selected to maximize the moisture signal in the tree growth. Middle-aged (~ 100–200 years old) and young (< 100 years) trees were specifically targeted for this study to avoid extremely narrow rings that were difficult to distinguish in older specimens. Core samples were placed in a Soxhlet apparatus and washed for approximately 24 h with 99% ethanol to remove resins and extractives, whereupon they were surfaced with a core microtome (Gärtner and Nievergelt 2010) and subsequently polished with 1200 grit sandpaper to fill cell voids with white wood dust. This two-tiered approach first ensures a flat surface and then maximizes the contrast, and thus the visibility of ring boundaries and cell structures prior to scanning. An Epson Expression 10000XL flatbed scanner, calibrated with SilverFast Ai scan software using the calibration target IT8.7/2, was used to produce digital images from the tree cores at 2400 dpi resolution (pixel size ~ 10 μm). The BI and partial ring-width measurements were carried out in the software CooRecorder version 9.4 (Larsson 2014). The BI measurements, defined here as absorbed light intensities, yield values positively correlated with wood density. Both the earlywood and latewood portions of the rings were analyzed, where the parameters average earlywood BI (EWBI) and maximum latewood BI (MXBI) corresponds to earlywood density and MXD, respectively, from X-ray based techniques (see table S1 in the supplement for CooRecorder program settings). Furthermore, we derived a third BI-parameter, defined as the difference between the MXBI and the EWBI parameters, called ΔBI (Björklund et al. 2014).

**Table 1:** Summary of the site and chronology characteristics

Site	Site ID	Lat	Long	Elevation (m.a.s.l.)	# of trees/series	Period > 5 series	Mean segment length (years)
Hornslandet	HORN	61.73	17.44	20	18/27	1742–2017	155
Myrkarby	MYRK	59.88	16.94	85	31/32	1701–2017	232
Ombo öar	OMBO	58.63	14.54	120	22/34	1722–2010	175
Gotland	GOTL	57.84	18.62	36	21/24	1798–2017	161
Fårhagsberget	FAHA	57.25	16.24	60	19/27	1731–2017	178

Visual cross-dating and measuring accuracy was checked with the program COFECHA (Holmes 1983). Note that the same cores and sample depths were used to produce the various parameter-level chronologies from each site. Thus, we did not omit from subsequent analyses accurately dated BI and partial ring width series exhibiting reduced correlations with the mean chronology. Because this study explores the potential of BI in a previously established, and now updated dataset, we focus our analyses on the high-frequency portion of variability.

We detrended the tree-ring data using 67-year cubic smoothing splines with a 50% frequency cut-off (Cook and Peters 1981). This detrending/standardization option preserves annual to decadal variability in the time-series, while eliminating longer-term frequency variations caused for example by the biological age trend or most of the potential heartwood/sapwood color changes (see Fig. 3). Site- and parameter-level chronologies were produced by averaging the dimensionless indices, and truncating the series where sample replication dropped below 5 series. A total of 30 chronologies of 6 different tree-ring parameters (including TRW, earlywood width (EW), latewood width (LW), EWBI, MXBI, and ΔBI) from 5 sites were included in the new dataset (Table 1).

### Data analysis

We calculated the Rbar (Wigley et al. 1984) and first-order autocorrelation (AR1) statistics on a site-by-site basis for individual tree-ring chronologies. The former statistic, computed as the average pair-wise correlation between individual detrended series, provides a measure of the strength of the common chronology signal. A principal component analysis (PCA) was performed on all chronologies over their common period (1798–2010) to evaluate common patterns of variability through time within the full network of chronologies. Additionally, PCA was performed separately on a parameter-level to summarize the regional common variability of each individual wood component. Time series of PCA scores of the first two principal components (PC1 and PC2) were retained for climate response analysis. Pearson correlation coefficients were computed to examine the relationship between the time series of the PCA scores and the monthly climate variables. The climate records included gridded monthly mean temperature and total precipitation data from the 0.5° by 0.5° gridded CRU TS 4.03 product (Harris et al. 2014), covering the period 1901–2018 and averaged over the region bounded by the latitude/longitude coordinates 57–62° N/14–19° E (Fig. 1). Additionally, climate responses of individual site-chronologies were analyzed using temperature and precipitation records from the CRU TS 4.03 grid-cell closest to each sample site (climate correlation for each individual site and parameter are provided in the supplement, Fig. S1). All climate data series were subjected to the same 67-year spline detrending as the tree-ring data to approximate the spectral properties of the predictor data. This was done to minimize bias in the correlation coefficients from potential multi-decadal and longer-term trends in the climate data, which have already been removed from the tree-ring data.

Calibration/verification experiments of the PC composites against the optimal season identified by the climate response analysis were conducted using simple linear ordinary least squares regression and an implementation of the split period (1901–1955 and 1956–2010 periods) calibration/verification approach. The test results were evaluated using squared Pearson correlation. Because we place the focus of our analysis on the higher frequencies, the standard reconstruction validation metrics Reduction of Error (RE) and Coefficient of Efficiency (CE) (Hanna 2007) are left without their normal evaluation efficacy. However, we still provide these statistics for reference in the supporting material (Table S2).

The long (1756–2018) quality-checked and homogenized instrumental climate records from the Stockholm meteorological station (Moberg and Bergström 1997; Moberg et al. 2002) were retained for further validation of the calibrated climate signal within the chronology network. Historical data of sea level pressure (SLP) was used as a surrogate for precipitation, since homogenized long-term (> 150 years) precipitation data are, as far as we know, not available from the region. The temporal stability of the tree-ring signal was assessed with running 31- and 51-year correlations between the PC composite chronologies and the Stockholm record in addition to the CRU TS 4.03 record used for calibration.

## Results

### Chronology signals

**Fig. 2:**
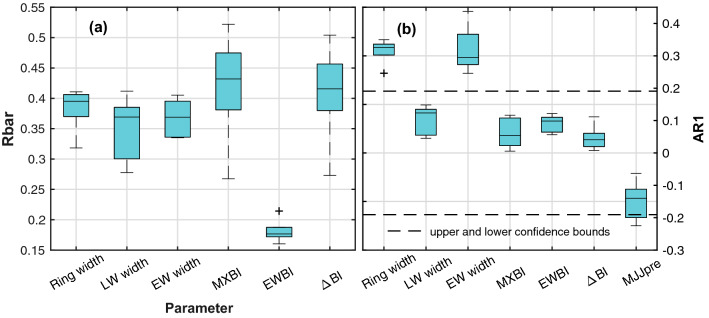
Boxplot summary of the **a** Rbar statistic and **b** first-order autocorrelation (AR1) for each tree-ring parameter. The AR1 coefficients are computed over the 1901–2010 modern interval. Also shown is the AR1 of the high-pass filtered warm season precipitation (MJJ pre). For definition of abbreviations, see Sect. 2.1

The median Rbar, based on five sites, is highest for the MXBI parameter (0.43), closely followed by the ΔBI parameter (0.42) (Fig. 2). Note, however, the large between-site spread for these two parameters, with Rbar coefficients ranging c. 0.27 to 0.52. The ring width, earlywood and latewood width parameters, conversely, tend to have a smaller range but lower median Rbar values at just below 0.4. EWBI has the weakest common signal with a median Rbar of 0.18. Significant first-order autocorrelations are only observed for ring width (median AR1 = 0.33) and earlywood width (0.29). For comparison purposes, we note that warm-season precipitation exhibits no significant persistence at interannual time-scales (AR1 = − 0.14 when filtered with a 67-year spline) (Fig. 2). The low and non-significant AR1 structure of LW width and the BI components (median AR1 = 0.04–0.12) are more consistent with instrumental climate target data than that of full ring-width and EW width.

**Fig. 3:**
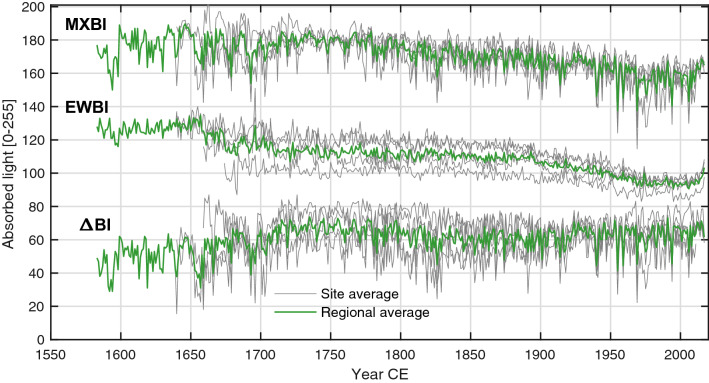
Raw non-detrended time series of absorbed blue light in the latewood (MXBI) and earlywood (EWBI) portions of the tree rings, and the derived ΔBI parameter. Site average (grey lines) and network average (green line) are shown

Average Pearson’s correlation coefficients between all chronology pairs are provided in Table 2, where all parameters, except EWBI, correlate significantly (*p* < 0.01) and positively with each other (*r* = 0.5–0.98 over the 1798–2010 period). In contrast, EWBI is negatively and mostly weakly correlated to ring width, EW width, MXBI and ΔBI. Figure 3 shows the non-detrended average BI chronologies. The EWBI parameter has less variability than the MXBI parameter, yet both parameters display trends that are in the same order of magnitude. The EWBI measurements will thus have less impact on the derived ΔBI parameter than MXBI, explaining why the correlations between the ΔBI parameter and MXBI are much stronger and less variable (*r* = 0.90–0.96) than the ones between ΔBI and EWBI (*r* = − 0.50 to − 0.17).

**Table 2:** Chronology correlation matrix over the common 1798–2010 period

	Ring width	LW width	EW width	MXBI	EWBI
Ring width					
LW width	0.80 ± 0.06				
EW width	0.96 ± 0.01	0.60 ± 0.06			
MXBI	0.71 ± 0.05	0.81 ± 0.04	0.57 ± 0.06		
EWBI	− 0.25 ± 0.10	0.07 ± 0.06	− 0.37 ± 0.08	0.03 ± 0.06	
ΔBI	0.76 ± 0.04	0.74 ± 0.06	0.67 ± 0.06	0.94 ± 0.03	− 0.32 ± − 0.12

Correlations are computed separately for each site. The table provides the average correlation across the network and its standard deviation

**Fig. 4:**
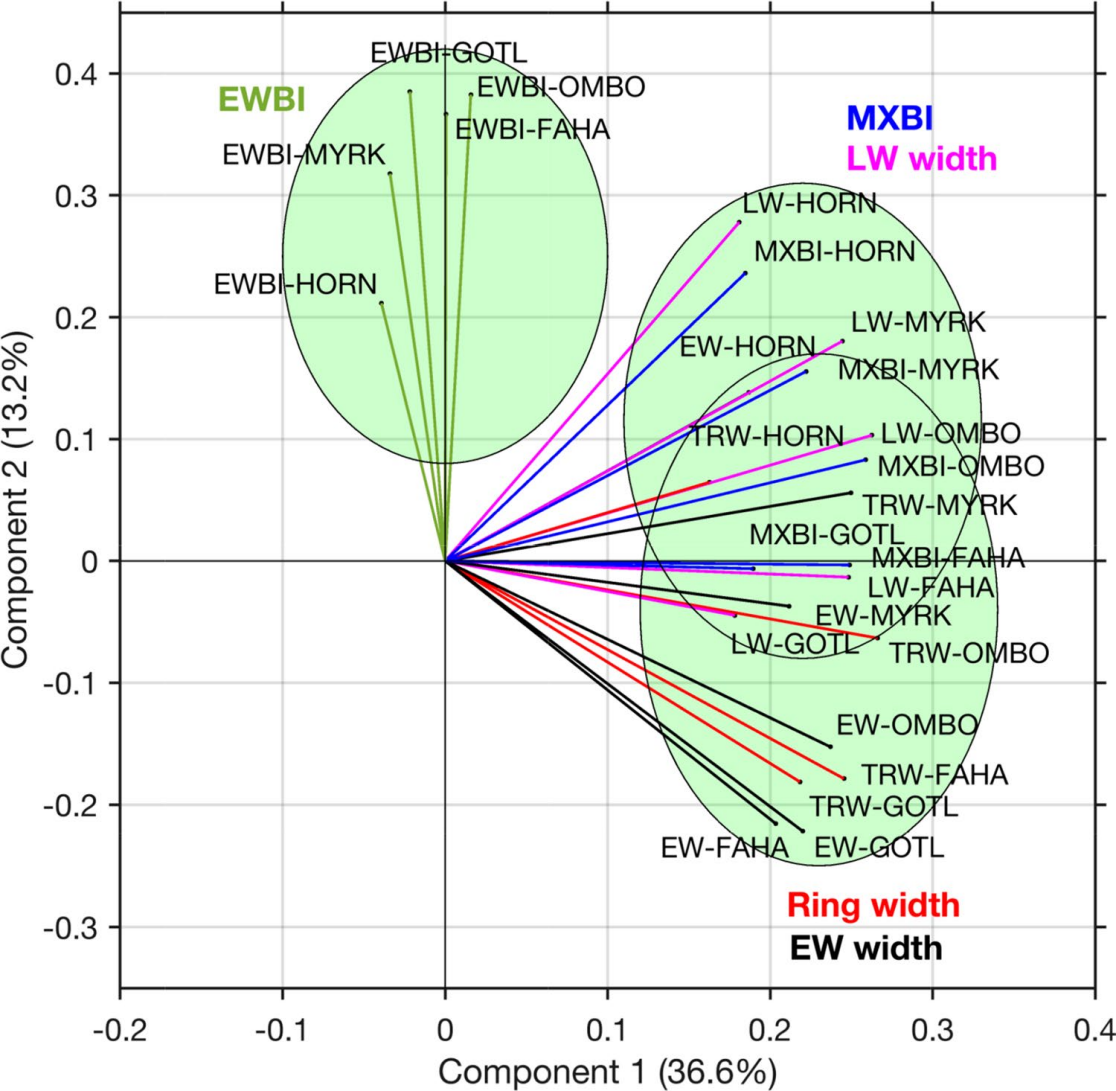
Biplot of the first two principal components of the PCA performed over the common 1798–2010 period on the multi-parameter collection from the five sites in Sweden. Identified parameter cohorts are highlighted in green. The color of the vectors corresponds to the different parameters (green lines—EWBI; blue—MXBI; pink—LW width; red—TRW; black—EW width). The first two components together represent nearly 50% of the total variation

To further examine the relationships between the various tree-ring parameters, a PCA was performed on all chronologies over their common period (1798–2010). The first two modes of PCA were analyzed as they represent the major variance in the network, explaining 36.6% and 13.2% of the total variability, respectively. Figure 4 gives the PCA biplot (Gabriel 1972). All ring width parameters and MXBI load positively and more or less equally strongly on the first component. However, the LW width and BI components partially separates from the ring width and EW width cluster, and the two cohorts appear in separate quadrants in the PCA biplot. Moreover, ordination of the two components shows a complete separation of EWBI from the rest of the chronologies, with the former having negligible influences on PC1 yet strong and positive loadings on PC2. Ring width and EW width share a negative loading on this axis and are thus negatively correlated with EWBI, as indicated by the ~ 130° angle between respective vector cohort. These results are indicative of a pronounced common signal, but also suggest the presence of distinct triple-signal patterns in the dataset. A climate sensitivity analysis was conducted to disentangle this signal structure and further explore the inter-site and inter-parameter consistency of variability (see Sect. 3.2).

### Climate signals

**Fig. 5:**
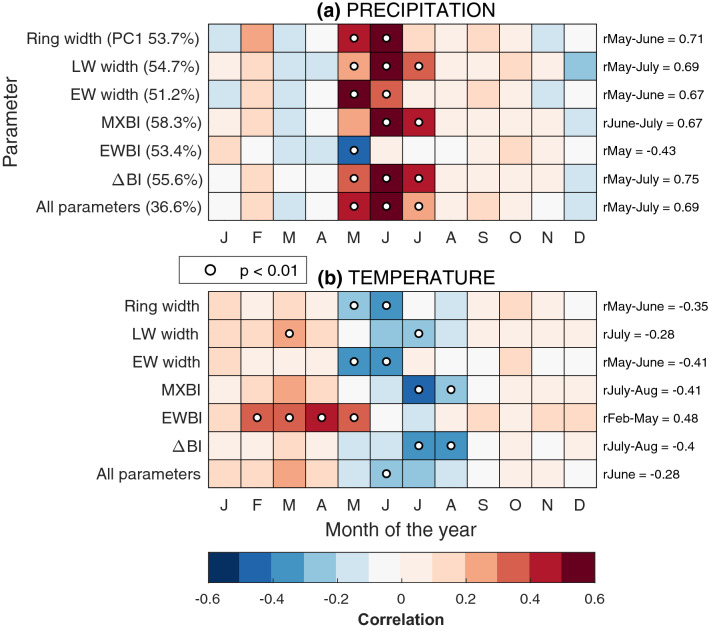
Simple linear correlations with CRU TS 3.2 monthly **a** precipitation and **b** temperature and the PC1 scores for each tree-ring parameter. Correlations are computed over the 1901–2010 interval using regional (57–62° N and 14–19° E), high-pass filtered, CRU TS 3.2 averages. The numbers in the parenthesis denote the amount of explained variation by the first PC component. Coefficients in the right of the plot are correlations with seasonally averaged climate variables

Pearson correlations between monthly climate variables and first principal component scores (PC1) of each tree-ring parameter in the full network are summarized in Fig. 5. The leading PC for each parameter accounts for 51–58% of the total variance, and approximately 37% when all sites and parameters are combined (Fig. 5), The climate correlation results for individual site chronologies can be found in the supplement (Fig. S1). No significant correlations were obtained for any of the PC2 scores (result not presented).

The correlations with both temperature and precipitation reveal consistent network-wide patterns, with minimal geographical differences over the study domain. The imprint of climate is clearly dominated by a moisture signal, which is evident through a strong relationship with precipitation in one or several months between May and July (Fig. 5). Notably, the different parameters respond to temporally consecutive, albeit overlapping, time windows within the growing season, in line with the successive formation and growth of the different tree-ring components. All parameters, except EWBI, display positive correlations with summer precipitation. The ring width and EW width are sensitive to early summer precipitation (May–June *r* = 0.67 and 0.71 for EW width and ring width PC1, respectively), while correlations between precipitation and LW width and MXBI are generally stronger towards mid-summer (June–July *r* = 0.67 for MXBI PC1 and May–July *r* = 0.69 for LW width PC1). The ΔBI displays, in general, an extended seasonal response window of up to three months, from May through July, with a slightly higher correlation (MJJ, *r* = 0.75 for ΔBI PC1).

Correlations with temperature (Fig. 5b) are weaker and less stable within the network, and mirror in most cases the correlations with precipitation (Fig. 5a). Most parameters, except EWBI, are negatively correlated (*r* ~ − 0.25 for PC1) with temperatures in one or several months in the May–August window, suggesting a moisture limitation caused by increased temperatures. Notably, stronger negative correlations are obtained when using monthly maximum temperatures rather than monthly average temperatures (results not shown). Moreover, tendencies toward a weakened warm-season thermal response are found with an increasing latitude of the sites (Fig. S1). These findings jointly reinforce the inference about water availability—drought being the main limiting factor for tree-growth in these high-latitude cool and well-drained environments. Except for EWBI, all parameters show weak positive lagged responses with prior years’ September rainfall (parameter PC1 mean *r* ~ 0.25). No significant lagged correlations were found with temperature in the prior year. The correlations of EWBI with climate variables stand out markedly from all other parameters. It displays a broad positive late winter- through- early summer temperature sensitivity (February–May *r* = 0.48 for PC1), and a seasonally narrow, yet geographically consistent, negative early summer (May) precipitation signal (*r* = − 0.43 for PC1).

**Fig. 6:**
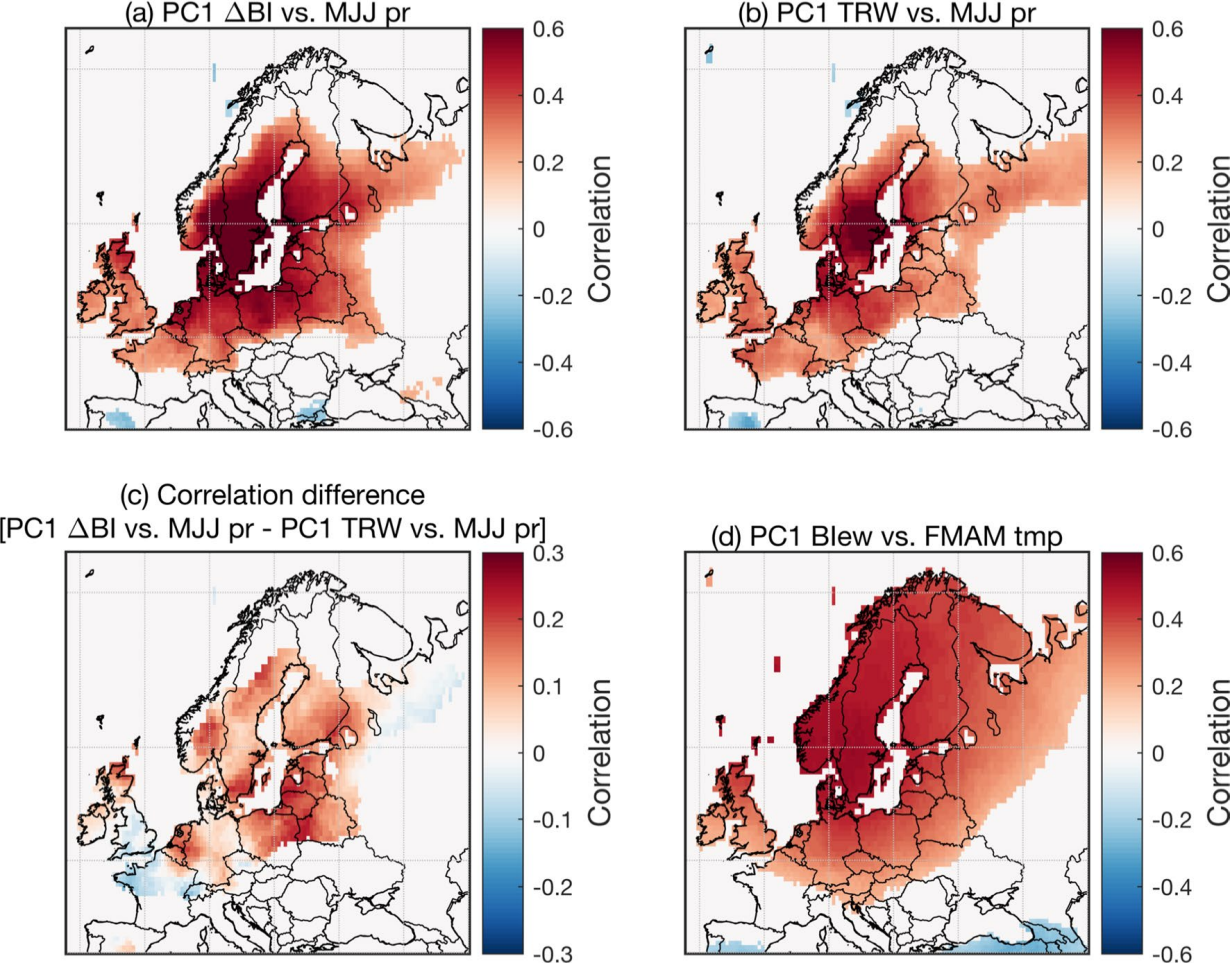
Field correlation between selected PC1 composite chronologies and gridded meteorological data from the CRU TS 4.03 product over the 1901–2010 period. **a** ΔBI and **b** ring-width (TRW) versus May–July precipitation, **c** the difference between the correlation fields shown in plots (**b**) and (**a**), **d** EWBI versus February–May temperature. Correlations are reported in color if significant (*p* < 0.05)

We compared the spatial extent of the precipitation signals expressed by BI and by ring-width, by computing spatial correlation fields between chronologies and gridded CRU TS 4.03 data (Fig. 6). The analysis was restricted to the regional ΔBI PC1 composite chronology, because it showed the strongest precipitation signal amongst the BI-based parameters (Fig. 5). The results for ΔBI reveal broad and significant (*p* < 0.05) correlations with May–July precipitation spatially (Fig. 6a), with significant correlations present over much of south-central Fennoscandia, central Europe and the British Isles in the 1901–2010 period. Particularly high correlations (*r* > 0.75) are, unsurprisingly, observed close to the sampling locations. Although the spatial response of the ring-width PC1 composite to MJJ precipitation is confined to roughly the same domain (Fig. 6b), correlations are substantially weaker (*r*-value differences as high as ~ 0.3) (Fig. 6c). We also assessed the spatial extent of the late winter to early summer temperature signal in the regional EWBI composite (Fig. 6d). We found it to be broad and statistically significant, with field correlation values of *r* > 0.4 over most of Fennoscandia.

### Time stability of the climate response

**Fig. 7:**
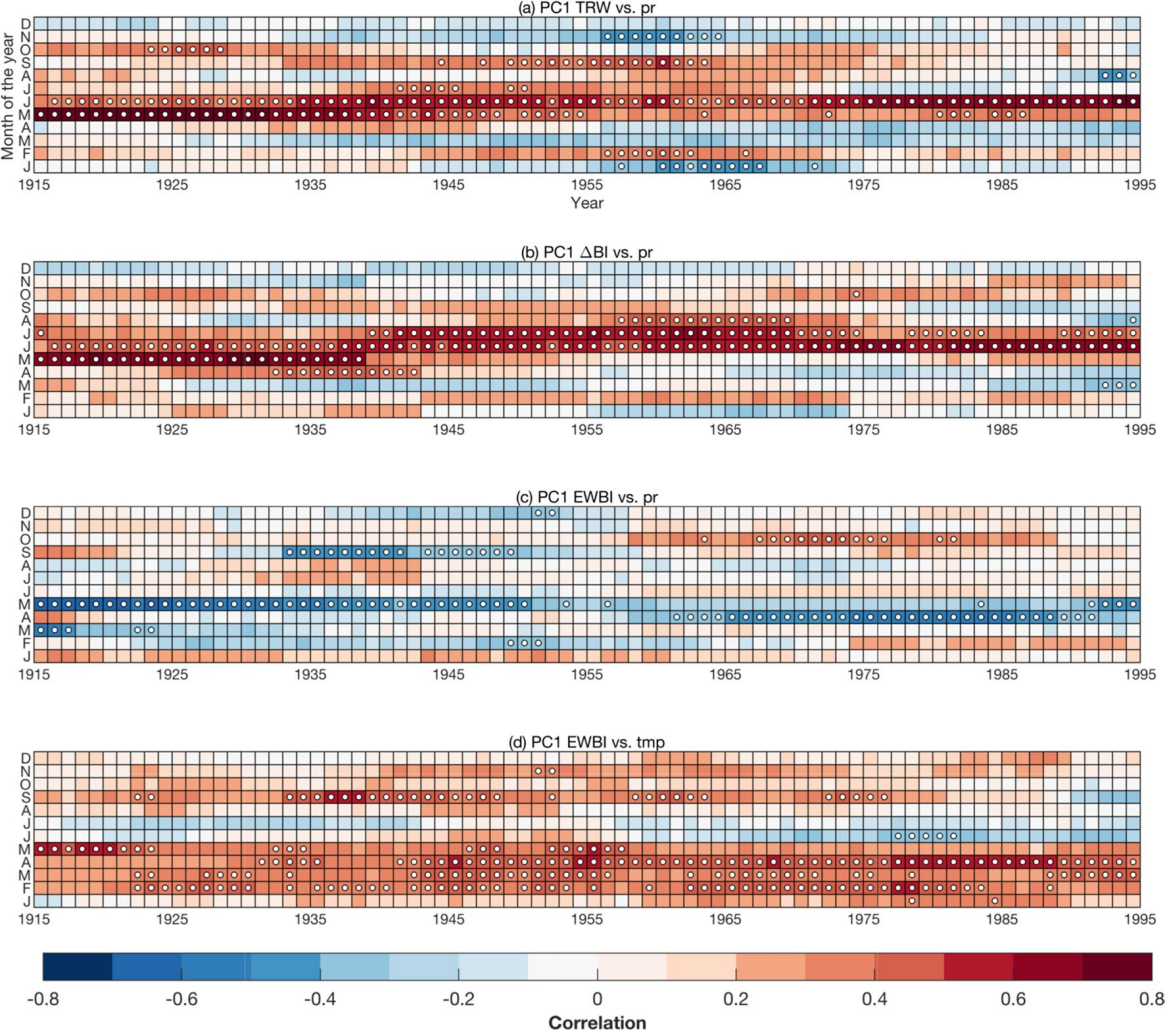
Moving 31-year window correlation over the 1901–2010 period between selected PC1 composite chronologies and gridded meteorological data from the CRU TS 4.03 product. **a** Ring-width (TRW) and **b** ΔBI versus precipitation, EWBI versus **c** precipitation and **d** temperature. Precipitation and temperature data have been high-pass filtered and averaged over the region bounded by the latitude/longitude coordinates 57–62° N/14–19° E

The temporal stability of the chronologies’ sensitivity to monthly climate was explored by means of moving window correlation analysis. Results for ring width, ΔBI and EWBI against the CRU TS 4.03 product (using a 31-year moving correlation window) are shown in Fig. 7, and against longer historical observations from Stockholm (using a 51-year moving correlation window) in Fig. S3 in the supplement. Both ring width and ΔBI display significant positive correlations with early summer rainfall throughout the full twentieth century; however, with slight changes in the seasonal timing of the climate response window and slight variation in the strength of the correlation coefficients (Fig. 7a, b). The ΔBI parameter display a step‐wise shift in the best seasonal window of the precipitation signal at around 1940s, shifting from May–June to June–July (Fig. 7b). Ring width, on the other hand, show a loss of sensitivity to May precipitaiton during recent decades (Fig. 7a). The precipitation and temperature signals in EWBI are comparatively weaker and more unstable. The negative early summer precipitation signal is shifting from May to April around 1950s (Fig. 7c). We also observe a slight weakening in the late winter through early summer temperature signal of EWBI in the first half of the twentieth century (Fig. 7d). Moving window correlations against Stockholm historical meteorological data over the 1798–2010 period show that all parameters have some degree of signal instability prior to the twentieth century, yet that ΔBI is the variable that displays the most stationary response over time (Fig. S3). The EWBI parameter largely loses its sensitivity to May precipitation (not shown), yet remains sensitive to late winter through early summer temperatures throughout the 1798–2010 interval (Fig. S3c).

### Climate reconstruction potential

Based on the climate sensitivity analysis (Sect. 3.2) and the tests of the uniformitarian principle (Sect. 3.3), we now evaluate the potential of the new tree-ring parameters in reconstructions of regionally-averaged warm-season precipitation and late-winter/early-summer temperatures in the high-frequency portion of the variability. We restrict the analysis to two variables: ΔBI and EWBI. ΔBI is selected because it contains the strongest, seasonally most broad, and temporally stable response to summer precipitation; EWBI because of its sensitivity to winter/spring temperatures—a rarely reconstructed climate variable and therefore particularly interesting from a paleoclimate perspective. For comparative purposes we also include ring width, as this is the tree-ring parameter that has most frequently been used as a predictor in hydroclimate reconstructions for Fennoscandia (Helama et al. 2009; Seftigen et al. 2013, 2017). The calibration experiments are performed separately for ΔBI and ring-width PC1 composites, due to the high collinearity among the two parameters.

The calibration/validation results for the different reconstruction are summarized in Table 3. We find that model calibration using ΔBI clearly provides the best estimate of warm-season (May–July) precipitation. The reconstruction model has consistent predictive power in the early (1901–1955) and late (1956–2010) calibration/verification subintervals, with model explained variance (*R*^2^) ranging from 54 to 58%. When calibrated over the entire 1901–2010 period, the regression model is able to account for 56% of the fractional variance in the instrumental precipitation data. In comparison, calibration of the tree ring width PC1 composite with May–June precipitation yield more unstable calibration/verification metrics (Table 3). Variance explained with the instrumental data peaks at 54% in the early part of the twentieth century, but drops to just 24% in the latter half of the century. The full interval ring width model captures just 37% of the variability in the observed MJJ precipitation, nearly 20 percentage points less than the ΔBI reconstruction. In contrast, statistically more robust results are found when calibrating the PC1 ring width composite with the shorter yet for this parameter optimal May–June target season, with *R*^2^-values of 0.51–0.55. For EWBI, calibration to February–May temperatures yielded more modest test statistics (*R*^2^ = 0.20–0.26) than the calibration experiments with ΔBI and ring width, yet with encouragingly stable reconstruction fidelity in the two calibration/verification subperiods.

**Table 3:** Calibration/validation results for reconstructions based on ring width (TRW), ΔBI and EWBI, respectively

		1901–1955 calibration		1956–2010 verification		Full 1901–2010 calibration	
Tree-ring parameter	Climate target	*r*	*R*^2^	*r*	*R*^2^	*r*	*R*^2^
PC1 TRW	MJ precipitation	0.74	0.55	0.69	0.47	0.71	0.51
PC1 TRW	MJJ precipitation	0.73	0.54	0.49	0.24	0.61	0.37
PC1 ∆BI	MJJ precipitation	0.76	0.58	0.73	0.54	0.75	0.56
PC1 EWBI	FMAM temperature	0.45	0.20	0.51	0.26	0.48	0.23

Experiments were performed over the 1901–1955, 1956–2010 and the full 1901–2010 periods. We used high-pass filtered precipitation and temperature data from the CRU TS 4.03 product, averaged over the region bounded by the latitude/longitude coordinates 57–62° N/14–19° E as a predictand

**Fig. 8:**
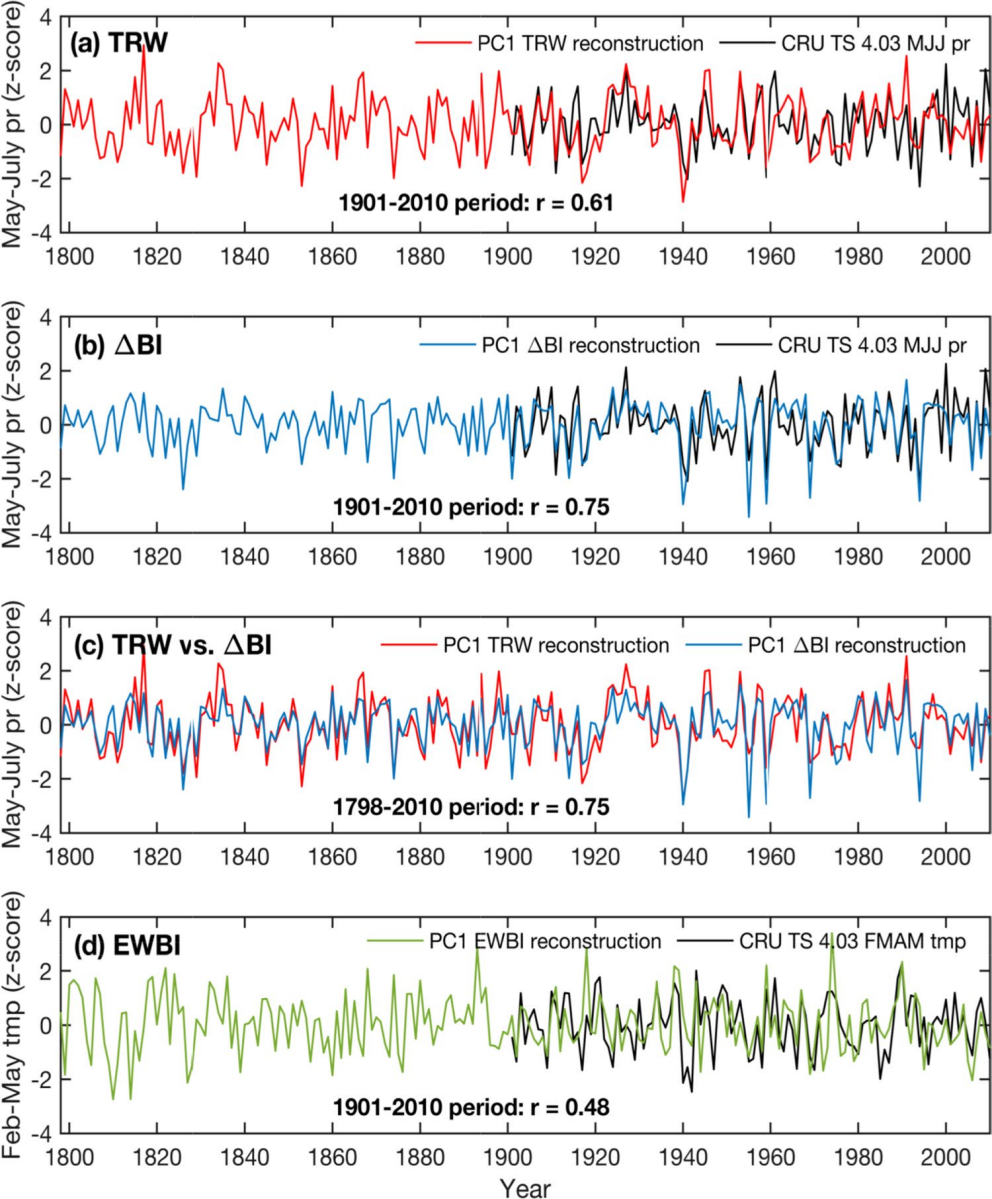
Scaled PC1 composite reconstructions and their target CRU TS 4.03 instrumental data. **a** Ring width- and **b** ΔBI-based MJJ precipitation reconstructions, and **c** comparison between these two reconstructions. **d** ΔBI-based February–May temperature reconstruction. Correlations between time-series are provided in the bottom of each plot. Note that data have been high-pass filtered and normalized to *z*-scores over the entire record length

**Fig. 9:**
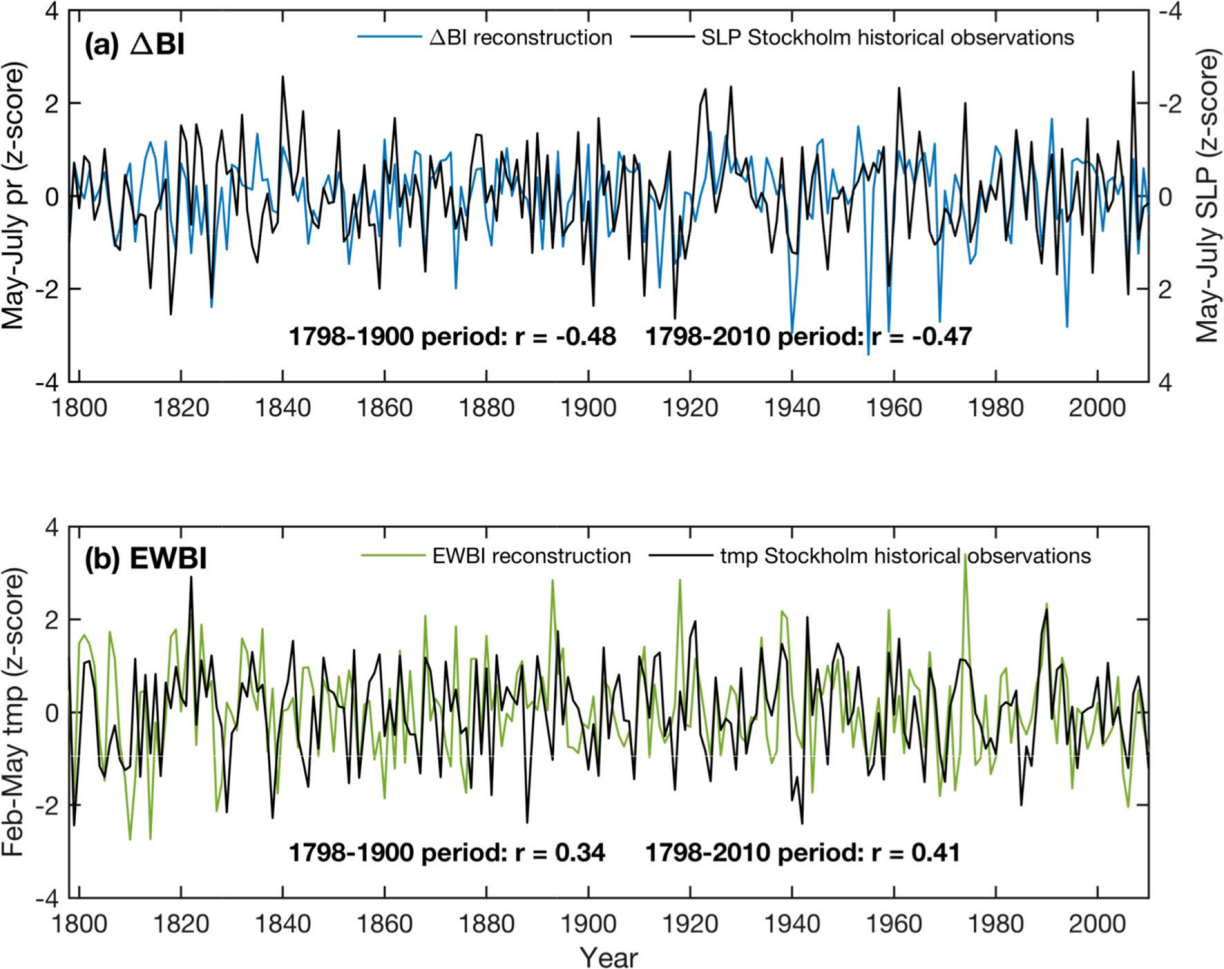
Time-series of the **a** ΔBI-based MJJ precipitation (pr) reconstruction and historical meteorological records of sea-level pressure (SLP); **b** EWBI-based FMAM temperature reconstruction and historical records of temperature (tmp) from the Stockholm meteorological station. Note that SLP explains less than 40% of the variability in summer precipitation in the region (Fig. S3). Data have been high-pass filtered and normalized to *z*-scores over the entire record length. Correlations between time-series are provided in the bottom of each plot

**Fig. 10:**
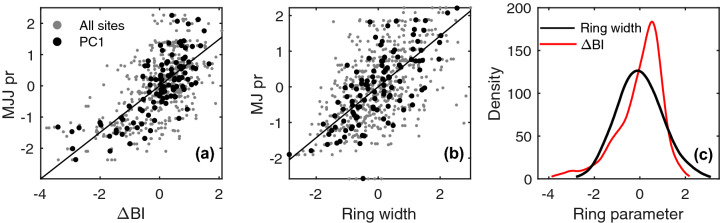
Scatter-plot comparisons between warm-season CRU TS 4.03 precipitation and **a** ΔBI and **b** ring width. Note that the regionally-averaged precipitation is normally distributed. **c** Kernel probability density functions of *z*-scored ring-width and ΔBI individual-site chronologies

The final parameter-based reconstructions are shown in Fig. 8. The ΔBI derived warm-season rainfall estimates are in close agreement with the CRU TS 4.03 observational precipitation data; however, we note that some of the dry extremes are overestimated in the reconstruction (potentially a measurement-related bias, see Fig. 10 and Sect. 4). The ΔBI and EWBI rainfall and temperature reconstructions are further compared with early data from the Stockholm meteorological station (Fig. 9), The long Stockholm records are independent from the CRU TS 4.03 dataset in the pre-1900 period, and thus provide additional validation of the calibrated signals. The correlations between the MJJ precipitation reconstruction and the fully independent historical SLP observations outside the calibration interval (1798–1900) is − 0.48, and over the entire common interval (1798–2010) − 0.47 (*p* < 0.001 in both cases), giving confidence that the reconstruction is temporally stable and reliable. The EWBI temperature reconstruction correlates at 0.41 (*p* < 0.001) with Stockholm temperatures over the full period of overlap, and 0.34 (*p* < 0.001) in the period withheld from calibration (1798–1900).

## Discussion

The current study provides the first comparison of the Blue Intensity (BI) measurements with ring width measurements from Scots pine growing in cool and humid albeit well-drained environments in Sweden. We show that all of these wood components are clearly and strongly correlated to precipitation during the warm-season, and that they are capable of providing fundamental information to advance our understanding of past hydroclimate variability. As a cold and relatively humid northern region, it risks a priori being overlooked in dendroclimatic studies of past droughts due to its rather benign hydroclimate regime (Ljungqvist et al. 2020b). Out of six investigated parameters, the ΔBI component appeared to capture the strongest precipitation signal. This permitted a cross-validated precipitation reconstruction capable of explaining well over half of regional-scale warm-season precipitation variance. We show that the ΔBI reconstruction not only improve the predictive skill of the ring width based model, with nearly a 20 percentage points increase in explained variance, but also allows for a broader seasonal window to be reconstructed, which is particularly important for the temporal stability of reconstructions (Frank et al. 2007).

Precipitation deficit and droughts are the main restriction of tree functioning, wood formation and growth in the cold and well-drained environments studied here. We found a strong link between low density of the latewood (i.e. a lower MXBI value) and dry, as well as hot, summer conditions. These results are in stark contrast to prior MXD/MXBI studies in the cooler and mountainous parts of Fennoscandia further north, where summer temperatures is identified as the main driver of latewood density (Esper et al. 2012; Fuentes et al. 2018; Linderholm et al. 2010,2015; Melvin et al. 2013), and where precipitation clearly plays a subordinate role (Seftigen et al. 2015b). While wood density in conifers is a function of tracheid dimensions (cell size and wall thickness) (Vaganov et al. 2006), the effect of cell size on wood density tends to dominate in the earlywood, whereas the density of the latewood component is generally more closely associated with variation in cell-wall thickness (Björklund et al. 2017; Yasue et al. 2000). Following this postulation, it is likely that the climate response pattern of latewood density revealed by our data is related to stomatal closure to prevent hydraulic failure thus reduced carbon assimilation rates in drier years (McDowell et al. 2008), resulting in resource deficiency in the wall thickening and lignification phase of latewood tracheids. This may explain why denser latewood predominates in years of increased latewood growth and favorable climate conditions. Moreover, in line with prior studies in warm (Camarero et al. 2014; Cleaveland 1986) and cold (Camarero et al. 2017) xeric environments, we found that wet conditions early in the growing season tend to promote earlywood width growth, and to reduce earlywood density. The precipitation–density coupling is likely connected to a drought-driven reduction in tracheid lumen size, and related decreases in hydraulic conductivity, radial growth and wood production (see Camarero et al. 2017 and references therein; Camarero et al. 2014). Notably, we also see a thermal dependence of the earlywood density, specifically that the formation of denser earlywood is correlated with warm late winter- through- early summer temperature (previously also observed in tropical conifers, Buckley et al. (2018)). The thermal control could be linked to earlier cambial resumption and prolonged duration and rate of metabolic processes (Rossi et al. 2006), leading to enhanced carbohydrate synthesis, increased lignification and cell wall synthesis of earlywood tracheids in response to a warm early growing season. Here, we can only hypothesize about these mechanisms, but continued efforts to shed more light on these issues would benefit from including measurements of the anatomical components of density (sensu Björklund et al. 2017).

Our results demonstrate the considerable potential of using BI to complement the currently available ring-width based hydroclimate reconstructions for the Fennoscandian region, and possibly also other regions with similar climate regimes. The ΔBI-parameter offers several advantages as a proxy. First of all, our data suggests a stronger common signal strength of ΔBI compared to ring width, meaning that fewer trees are required to provide a representative sample for calibration with climate data. Whether this holds true at longer (multi-decadal to multi-centennial) time-scales given the potential limitation of BI parameters at these frequencies (Björklund et al. 2014; Buckley et al. 2018; Rydval et al. 2014) remains, however, to be evaluated. We note also that a weaker common signal of the ΔBI parameter is not uncommon to find in other tree species and environments (e.g., Blake et al. 2020), and stress that this evaluation always should be exercised on a site-by-site basis. However, the ease and low cost of producing BI data does not pose a severe obstacle for increasing replication unless tree samples are simply not available. Secondly, the first-order autocorrelation of the ΔBI parameter is insignificant. That is, the autocorrelation of the ΔBI parameter is thus more consistent with the autocorrelation of the target instrumental precipitation data than with ring width. In fact, it has repeatedly been shown that ring width chronologies have strong biological persistence and higher autocorrelation than MXD (Büntgen et al. 2015; Esper et al. 2015; Ljungqvist et al. 2020a). Likewise, ring width dominated proxy collections tend to overestimate the ratio of low- to high-frequency variability in the instrumental records (Ault et al. 2013; Franke et al. 2013; Hartl-Meier et al. 2017).

Importantly, the ΔBI based warm-season rainfall reconstruction provides a substantial improvement over previous ring width reconstructions because it not only expands the reconstructed seasonal window, it is also able to explain a larger fraction of the variance of the instrumental record, as well as being less subjected to signal instabilities over time. In fact, a longer optimal target window allows for temporally less variable correlations between the tree ring chronology and the seasonally averaged climate target, as small shifts in seasonality will have a more pronounced implications when the target window is narrow (Frank et al. 2007). Moreover, using MXBI and EWBI and partial-width measurements, rather than ring width alone, can provide information about climate in different portions of the growing season, adding a finer level of detail at an intra-seasonal scale.

There are still many unresolved problems facing the application of BI. There may be trends and variability in these measurements that are not linked to climate, but rather to systematic color changes of the wood material (Björklund et al. 2014; Buckley et al. 2018; Rydval et al. 2014; Wilson et al. 2017), such as the abrupt heartwood/sapwood color transition, fungal staining or oxidation (Björklund et al. 2019). The heartwood/sapwood bias is apparent in the raw un-detrended BI time-series (Fig. 3), seen as an increasingly steep decline around 1900 CE subsequently neutralized around 1960. It arises because each raw measurement of MXBI and EWBI exhibit a more or less negative step in trend when transitioning from heartwood to sapwood, and that most heartwood/sapwood transitions of our sample material dates to between the late nineteenth century and mid-twentieth century. Because the heartwood/sapwood discoloration arguably is similar in the earlywood and latewood, it could be possible to neutralize the color difference by subtracting the EWBI from the MXBI to derive the ΔBI (Björklund et al. 2014). Nevertheless, with the dynamic detrending implemented in this study, the systematic and non-climate related trends should be neutralized in all parameters, allowing us to identify unbiased climate imprints in the tree-ring data at high frequencies. The discoloration problems would however require more careful scrutiny when attempting to preserve lower-frequency signals in the BI time-series, e.g. at centennial to multi-centennial time-scales. In this context, the ΔBI parameter appears even more attractive than has already been shown in the statistical criteria tested in this study.

Additional biases using basic BI techniques include the relatively low measurement resolution (Björklund et al. 2019). The MXBI of climate stressed conifers may in some instances be represented by only a few cells in the radial direction (Vaganov et al. 2006). If the measurement resolution is coarser than the radial extension of the “true” MXD or MXBI area, then these parameters will be deflated and increasingly dependent on the latewood width (see Fig. S4 in the supporting material). If we consider for our material that MXD and ring width have a strong and true relationship, very narrow rings naturally are accompanied by very low MXBI values. A potential measurement bias postulate that narrow rings will have even lower MXBI values than expected. This is probably a reason for the observed skewed distribution of the MXBI values in our collection (Fig. 10c), and it may explain the tendencies to a non-linear relationship between MXBI and precipitation (Fig. 10a). Thus, even though our results are very encouraging for hydroclimate reconstructions of the region, there appears to be additional room for improvement using highly resolved techniques such as X-ray densitometry or even quantitative wood anatomy. The advantage using anatomical density of course includes a more in-depth understanding of the derived signals, where climate signals are expected to be more distinct from ring widths, but also, the complete removal of any heartwood sapwood bias (Björklund et al. 2019, 2020).

## Conclusions

In this paper we explore if an improved inference of past hydroclimate can be obtained using Blue Intensity (BI) and partial ring width measurements from a network of cool and drought-prone *Pinus sylvestris* sites in southern and central Sweden. By comparing the different width- and BI-parameters and their relation with climate, we find great potential of using wood density also in well-drained areas with relatively benign climate. These novel findings provide a significant empirical foundation in the high-latitude Northern Hemisphere, where presently few truly moisture-sensitive high-resolution proxy data exist (Linderholm et al. 2018). Moreover, the results are of particular importance for Fennoscandia, where the available long moisture-sensitive proxy records possess an unrealized potential using only ring width (e.g., see the tree-ring collection in Cook et al. 2015). We explicitly draw attention to the dendroclimatic potential of ΔBI-derived reconstruction using our approach, explaining > 55% of the variance in warm season, high-pass filtered, precipitation. This is an improvement of nearly 20 percentage points in explained variance in the predictive skill of the ring width based precipitation reconstruction. Thus, by developing wood densitometric chronologies there is a great potential to produce the most robust tree-ring reconstructions of hydroclimate so far available in the Fennoscandian region.

This research has focused on one species and a fairly limited geographical portion of Fennoscandia. More research is needed to determine if our findings can be generalized to additional conifer species and other high-latitude cool-dry sites. Moreover, we recommend that future work should be focused on increasing the temporal extension of chronologies and to implement more highly resolved measurement techniques to maximize, and to more fully understand the imprinted climate signals, as well as minimize measurement biases.

## Electronic supplementary material

Below is the link to the electronic supplementary material.

Supplementary file1 (DOCX 19572 kb)
