# Supplementary material for: Using Blue Intensity from drought-sensitive Pinus sylvestris in Fennoscandia to improve reconstruction of past hydroclimate variability
Source: Clim Dyn. 2020 May 13;55(3):579–94. doi: 10.1007/s00382-020-05287-2 (PMC7370983; doi:10.1007/s00382-020-05287-2)
Supplement: Supplementary file 1 — Supplementary file1 (DOCX 19572 kb) [file 382_2020_5287_MOESM1_ESM.docx]

**Supporting information**

***Figure S1:*** *Correlation between individual site chronologies and CRU TS 4.03 monthly mean temperature and total precipitation from the nearest grid point. Correlation analysis has been performed over the 1901-2010 interval, common to all chronologies. Both tree-ring and climate data have been filtered with a 67-year spline prior to analysis. Significant correlations (p<0.05) are outlined. See table 1 for site abbreviations.*

***Figure S2:*** *Moving 51-year correlation over the 1798–2010 period between selected PC1 composite chronologies and high-pass filtered historical meteorological data from the Stockholm meteorological station. a) ring-width (TRW) and b)* Δ*BI versus sea-level pressure (SLP*; *is used here as a surrogate for precipitation), c) EWBI versus temperature. Significant (p < 0.05) correlations are outlined. Note that air pressure explains less than 40% variability in summer precipitation in the region (Fig. S3).*


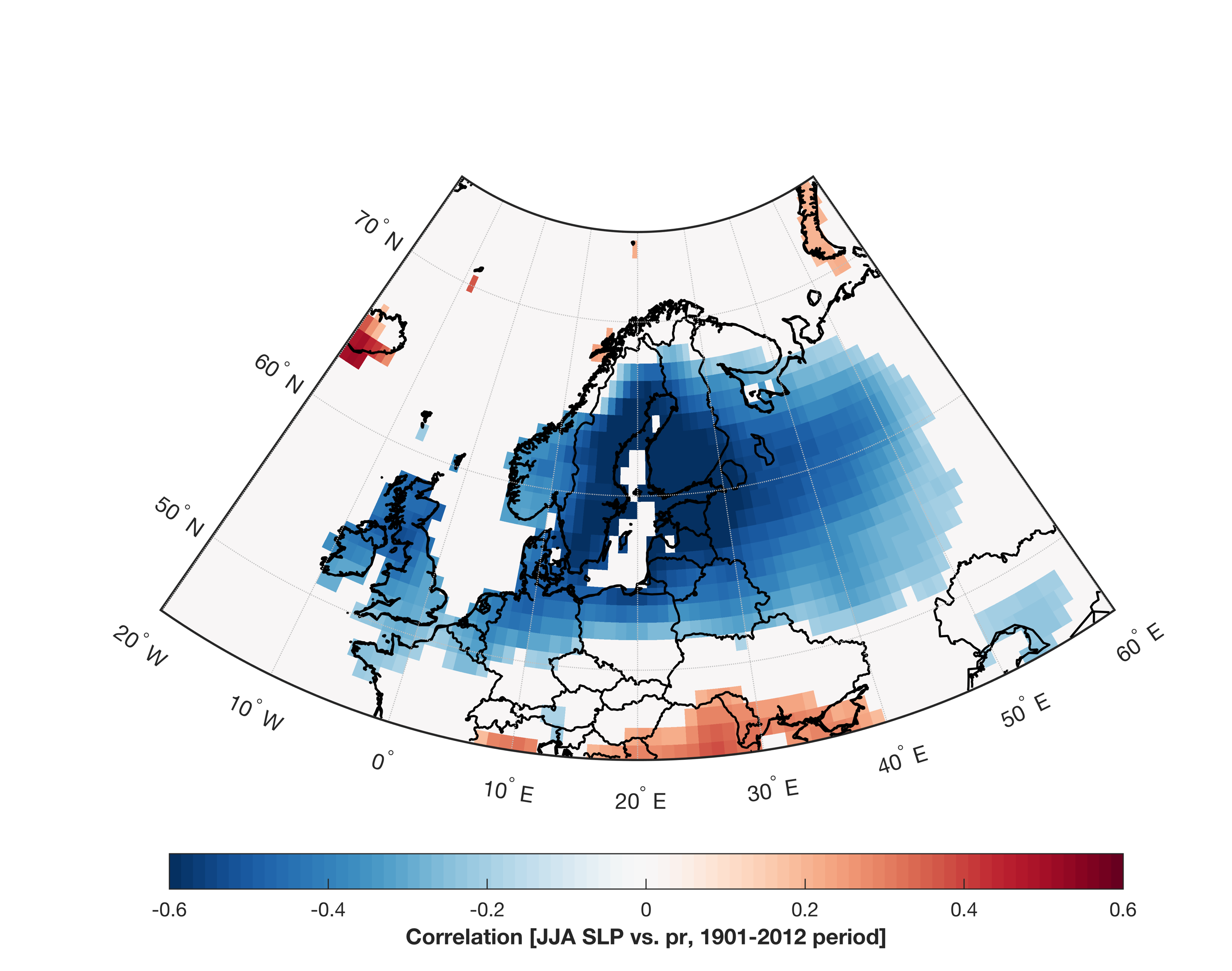


***Figure S3:*** *Point-by-point correlation between sea level pressure measured at the Stockholm historical observatory and precipitation from the CRU TS 4.03 product. Both datasets are averaged over the June-August season. Correlations are reported in color if significant (p<0.05).*

***Figure S4:*** *Scatterplots between raw un-detrended values of latewood width (LW) and BI-measurements in latewood (MXBI) from five sites (see table 1 for site ID). The plots are based on measurements from individual cores.*

***Table S1:*** *CooRecorder program settings used to generate MXBI and EWBI.*

| **Parameter** | **w** | **wLimFact** | **f** | **d** | **k** | **Select data by:** | **% of the dark or light wood to use for color data** |
| --- | --- | --- | --- | --- | --- | --- | --- |
| **MXBI** | 80 | 10 | 5 | 50 | 0.30 | “Mean of sorted pixels” | 15 |
| **EWBI** | 80 | 10 | -2 | 500 | 0.10 | “Mean of slices” | 80 |

***Table S2:*** *Calibration/validation results for reconstructions based on ring width (TRW), ΔBI and EWBI, respectively. Experiments were performed over the 1901–1955, 1956–2010 and the full 1901–2010 periods. We used high-pass filtered precipitation and temperature data from the CRU TS 4.03 product, averaged over the region bounded by the latitude/longitude coordinates 57–62° N/14–19° E as a predictand.*

|  |  | **1901–1955 calibration** | | | | **1956–2010 verification** | | | | | **Full 1901–2010 calibration** | | |
| --- | --- | --- | --- | --- | --- | --- | --- | --- | --- | --- | --- | --- | --- |
| **Tree-ring**  **parameter** | **Climate target** | **r** | **R^2^** | **RE** | **CE** | **r** | **R^2^** | **RE** | **CE** | **r** | | **R^2^** |  |
| PC1 TRW | MJ precipitation | 0.74 | 0.55 | 0.46 | 0.46 | 0.69 | 0.47 | 0.54 | 0.54 | 0.71 | | 0.51 |  |
| PC1 TRW | MJJ precipitation | 0.73 | 0.54 | 0.24 | 0.24 | 0.49 | 0.24 | 0.54 | 0.53 | 0.61 | | 0.37 |  |
| PC1 ΔBI | MJJ precipitation | 0.76 | 0.58 | 0.53 | 0.53 | 0.73 | 0.54 | 0.58 | 0.57 | 0.75 | | 0.56 |  |
| PC1 EWBI | FMAM temperature | 0.45 | 0.20 | 0.27 | 0.26 | 0.51 | 0.26 | 0.20 | 0.20 | 0.48 | | 0.23 |  |
